# Commentary: Primary Language as a Predictor of Perioperative Opioid Consumption and Clinical Outcomes in Breast Reconstruction

**DOI:** 10.1177/22925503261482099

**Published:** 2026-09-08

**Authors:** Caroline Esmonde-White, Tyler Safran, Joshua Vorstenbosch

**Affiliations:** 1 Division of Plastic, Reconstructive, and Aesthetic Surgery, Department of Surgery, 54473 McGill University, Montreal, Canada; 2 Division of Plastic and Reconstructive Surgery, Surgery Department, 54473 McGill University Health Centre, Montreal, Canada

Effective communication between healthcare providers and patients is critical, especially in the perioperative setting.^1,2^ Patients with limited proficiency in the primary language may face barriers, including difficulties in accessing care, providing informed consent, and understanding post-operative instructions.^2^ In this study, Wang et al investigated differences in clinical outcomes between English-speaking patients and those with limited proficiency in English (LEP) when undergoing autologous and implant-based breast reconstruction.^3^

The authors performed a retrospective cohort study of 1706 patients at a single institution and grouped patients by their recorded primary language. Outcome measures were opioid consumption and perioperative complications, such as seroma, hematoma, surgical site infection (SSI), unexpected reoperation, readmission, and ED visits within 90 days. Notably, there was an association between non-English speakers and lower rates of total and post-operative opioid consumption in the overall sample, as well as in both the autologous breast reconstruction and implant-based reconstruction (IBR) groups. Post-operative complication rates were also found to differ between English-speaking and LEP groups, including sub-analysis demonstrating higher rates of seroma in the LEP group in the overall sample and higher rates of SSI in the LEP group undergoing IBR. Taken together, these results indicate that differences do exist between English and LEP groups undergoing breast reconstruction in terms of perioperative pain management and complication profile.

The importance of this study is in highlighting disparities in breast reconstruction outcomes associated with patient proficiency in the primary language. Prior studies have identified that patients who do not speak the primary language were less likely to undergo breast reconstruction compared to English speakers, indicating that disparities exist both pre- and post-operatively.^4-7^ Further studies should aim to analyze and address root causes to reduce differences rooted in language proficiency. Possible explanations provided in this study include provider difficulty in assessing pain levels, patient reluctance to present with early-stage complications, and lack of understanding of post-operative instructions. This emphasizes the need to bridge the communication gap with LEP patients using adequate translation and understanding of cultural values and norms. This is particularly salient in the field of breast reconstruction due its deeply personal and subjective nature.^6^

Future studies may also investigate if these differences exist across institutions or in settings with primary languages other than English. Other future directions include powering analysis to examine differences with pre- or sub-pectoral reconstruction and the extent of nodal surgery, as these technical considerations may impact the need for analgesia and post-operative outcomes.

In conclusion, we commend the authors on a thought-provoking examination of how English-language proficiency may affect opioid consumption and perioperative complications in the setting of autologous and implant-based breast reconstruction. We hope that the findings of this study will guide surgeons to consider primary language proficiency when managing perioperative breast reconstruction pain and complications.
